# Novel Escape Mutants Suggest an Extensive TRIM5α Binding Site Spanning the Entire Outer Surface of the Murine Leukemia Virus Capsid Protein

**DOI:** 10.1371/journal.ppat.1002011

**Published:** 2011-03-31

**Authors:** Sadayuki Ohkura, David C. Goldstone, Melvyn W. Yap, Kate Holden-Dye, Ian A. Taylor, Jonathan P. Stoye

**Affiliations:** 1 Division of Virology, MRC National Institute for Medical Research, London, United Kingdom; 2 Division of Molecular Structure, MRC National Institute for Medical Research, London, United Kingdom; Fred Hutchinson Cancer Research Center, United States of America

## Abstract

After entry into target cells, retroviruses encounter the host restriction factors such as Fv1 and TRIM5α. While it is clear that these factors target retrovirus capsid proteins (CA), recognition remains poorly defined in the absence of structural information. To better understand the binding interaction between TRIM5α and CA, we selected a panel of novel N-tropic murine leukaemia virus (N-MLV) escape mutants by a serial passage of replication competent N-MLV in rhesus macaque TRIM5α (rhTRIM5α)-positive cells using a small percentage of unrestricted cells to allow multiple rounds of virus replication. The newly identified mutations, many of which involve changes in charge, are distributed over the outer ‘top’ surface of N-MLV CA, including the N-terminal β-hairpin, and map up to 29 A^o^ apart. Biological characterisation with a number of restriction factors revealed that only one of the new mutations affects restriction by human TRIM5α, indicating significant differences in the binding interaction between N-MLV and the two TRIM5αs, whereas three of the mutations result in dual sensitivity to Fv1^n^ and Fv1^b^. Structural studies of two mutants show that no major changes in the overall CA conformation are associated with escape from restriction. We conclude that interactions involving much, if not all, of the surface of CA are vital for TRIM5α binding.

## Author Summary

Host restriction factors such as TRIM5α are important for preventing cross species transmission of a variety of retroviruses. They act to block viral replication but their mode of virus recognition is poorly understood. To address this question we have developed a procedure for isolating viruses that replicate in the presence of restriction factors. Analysis of these viruses shows that individual mutations across the entire surface of the viral capsid molecule can relieve restriction. Escape from TRIM5α of one species does not necessarily lead to escape from another. It seems likely that restriction factor recognition involves extensive weak contacts between factor and virus. We suggest that this represents an important design feature in a system that recognizes multiple pathogens.

## Introduction

Mammalian cells show different susceptibilities to retrovirus infection. Cells from mice exhibit different susceptibility to murine leukaemia virus (MLV) dependent on their genetic backgrounds; cell lines from different primates show defined patterns of lentiviral replication. In many cases, these variations are due to the presence of cellular proteins referred as restriction factors. The susceptibility to MLV infection in mouse cells is determined by a restriction factor called Fv1; different alleles of Fv1 restricting different strains of MLV. Fv1^n^ restricts B-tropic MLV (B-MLV) but not N-tropic MLV (N-MLV) infections, whereas Fv1^b^ does the reverse [1], [2], [3]. In primate cells, lentivirus and N-MLV are restricted by a second restriction factor, TRIM5α [4], [5], [6], [7], [8]. TRIM5 is a member of a large family of tri-partite motif proteins that contain RING, B-box and coiled-coil motifs, sometimes referred to collectively as an RBCC domain [9], [10]. The α isoform of TRIM5 (TRIM5α) also has an additional large B30.2 or PRYSPRY domain at its C-terminus [11], [12], important for recognizing the viral target [13], [14], [15], [16]. Both Fv1 [17], [18] and TRIM5α [5], [19] target the viral capsid protein (CA), blocking retrovirus replication after viral entry into the target cell. Restriction by TRIM5α, however, is clearly distinguished from that by Fv1 in that TRIM5α-mediated restriction normally occurs before or during reverse transcription [5], [20], whereas Fv1-mediated restriction occurs after the completion of reverse transcription but prior to viral integration [21], [22].

One of the most surprising features of TRIM5α restriction is the ability of TRIM5αs from different species to recognize and restrict multiple unrelated viruses often across different retroviral genera. For example, TRIM5α from the cotton-top tamarin can recognize at least one member from each of the lentivirus, gammaretrovirus, betaretrovirus and spumavirus genera and rhesus monkey (rh) TRIM5α will restrict both HIV-1 and N-MLV [23], [24], [25]. Although it is clear that Fv1 and TRIM5α target viral CA, in the absence of structural information the recognition process remains poorly defined. However, it appears likely that single-site binding is weak and that a productive interaction required for restriction is multivalent and dependent on the spacing of CA monomers/hexamers in the viral core [26], [27], [28].

One approach to investigating the problem of restriction specificity has been through genetic characterisation of naturally occurring or experimentally induced variants in CA that alter virus tropism [29]. In this way amino acid residues involved in the interaction with restriction factors have been identified. In the case of Fv1-mediated restriction of MLV, it is known that several surface-exposed amino acids in the region between α-helices 4 and 6 in CA, which include amino acids at positions 82, 92–94, 110, 114 and 117, can change the tropism of MLV [30]. Among these positions, the amino acid at position 110 is the main determinant, since substitution of amino acid at position 110 (Arg for N-tropic and Glu for B-tropic MLV) can switch the tropism of MLV [31]. Structural studies show that amino acids in positions 110, 114 and 117 are located in α-helix 6, residue 82 is in the loop between α-helices 4 and 5, and amino acids 92–94 are located in α-helix 5 [32]. Therefore, the region between α-helices 4 and 6 might be considered a ‘Fv1-binding pocket’ [27]. A number of amino acid substitution experiments have also suggested that this α4–α6 region may also contribute to TRIM5α-mediated restriction of N-MLV. These include an R110E mutation that confers resistance of N-MLV to restriction by human (hu) TRIM5α and an E110R mutation in B-MLV that results in susceptibility to restriction by huTRIM5α [19]. Other substitutions in CA at positions 82, 114 and 117 alter the TRIM5α species range able to restrict B- and N-MLV [8] and domain-swap experiments suggested that the equivalent region in HIV and other lentiviruses also contains the host-range determining segments [33], [34], [35], [36].

In this study, replication competent N-MLV was passaged in rhTRIM5α-positive cells to search for escape mutations in sites important for restriction and in the context of a natural infection. We isolated a number of N-MLV escape mutants, including substitutions at the previously identified positions 82 and 114. However, of particular interest is a new class of mutations including G8D, L10W and V116D that were discovered. They map outside the putative Fv1 binding pocket and display subtle structural changes. In these instances, escape from restriction by rhTRIM5α was conferred, but restriction by huTRIM5α was not affected. These observations have defined new regions of N-MLV CA that control virus growth in the presence of TRIM5α, revealed that the restriction factor-CA interface is larger than previously thought and shown that subtle changes in CA structure can have profound effects on TRIM5α susceptibility.

## Results

**Figure 1:**
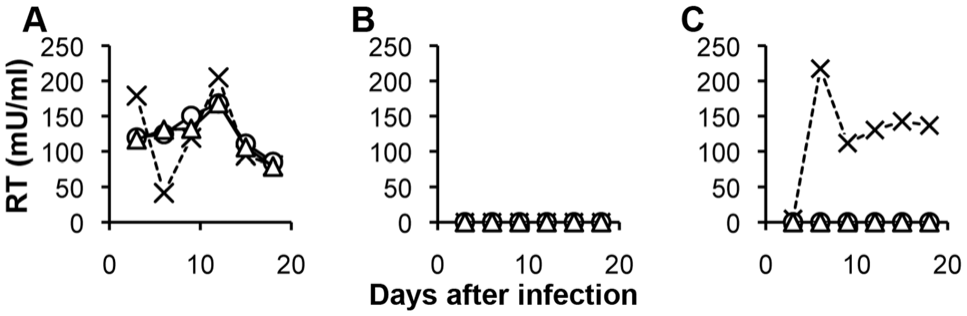
Growth of replication competent N-MLV in human and rhesus macaque TRIM5α-expressing MDTF cells. TRIM5α-negative MDTF cells (A), human TRIM5α-positive MDTF cells (B) and rhesus macaque TRIM5α-positive MDTF cells (C) were inoculated with different dilutions of replication competent Stock A N-MLV (undiluted, cross; 10-fold diluted, triangle; 100-fold dilution, circle). Infected cells were passed twice a week for eighteen days monitoring RT activity in the culture supernatant. Similar results were obtained from two independent experiments, one example is shown.

In an initial attempt to isolate MLV variants resistant to TRIM5α-mediated restriction, *Mus dunni* tail fibroblasts (MDTF), MDTF expressing YFP and huTRIM5α (MDHu) or rhTRIM5α (MDRh) were inoculated with different dilutions of replication competent N-MLV (Stock A) and the infected cells were passed repeatedly. On each passage, the culture supernatant was harvested and reverse transcriptase (RT) activity in the filtered culture supernatant measured (Figure 1). After only three days high RT levels were measured in restriction factor-negative cells, even when the target cells were initially infected with low titre (1∶100 diluted virus) (Figure 1A). When restriction factor-negative cells were infected with the high titre undiluted virus, the RT activity decreased sharply at 6 days post infection (d.p.i.) but later recovered, due to virus-mediated cytopathic effects. In cells expressing huTRIM5α N-MLV replication was restricted completely, even when undiluted virus was inoculated (Figure 1B). By contrast, cells expressing rhTRIM5α did not prevent replication of high titre virus, and, although the time of RT release was delayed, maximal levels were similar to those seen with MDTF (compare Figure 1C and Figure 1A). No virus was recovered from MDRh infected with 1∶10 or 1∶100 diluted N-MLV (Figure 1C).

Taken together, these data imply (a) huTRIM5α restriction of N-MLV is more potent than that of rhTRIM5α and (b) rhTRIM5α restriction can be overcome by high levels of wild-type N-MLV in a rapid manner, presumably by saturation. However, under these conditions, an extended period of virus replication against a background of restriction does not seem to occur leaving little opportunity for escape mutants to arise. To maximize the chances of generating and recovering escape mutants we decided to focus on searching for escape mutants from rhTRIM5α using low input virus to avoid restriction factor saturation. To allow continued virus replication under selective conditions a mixture of MDTF and MDRh was infected.

### Generation and selection of N-MLV escape mutants from rhesus TRIM5α

**Figure 2:**
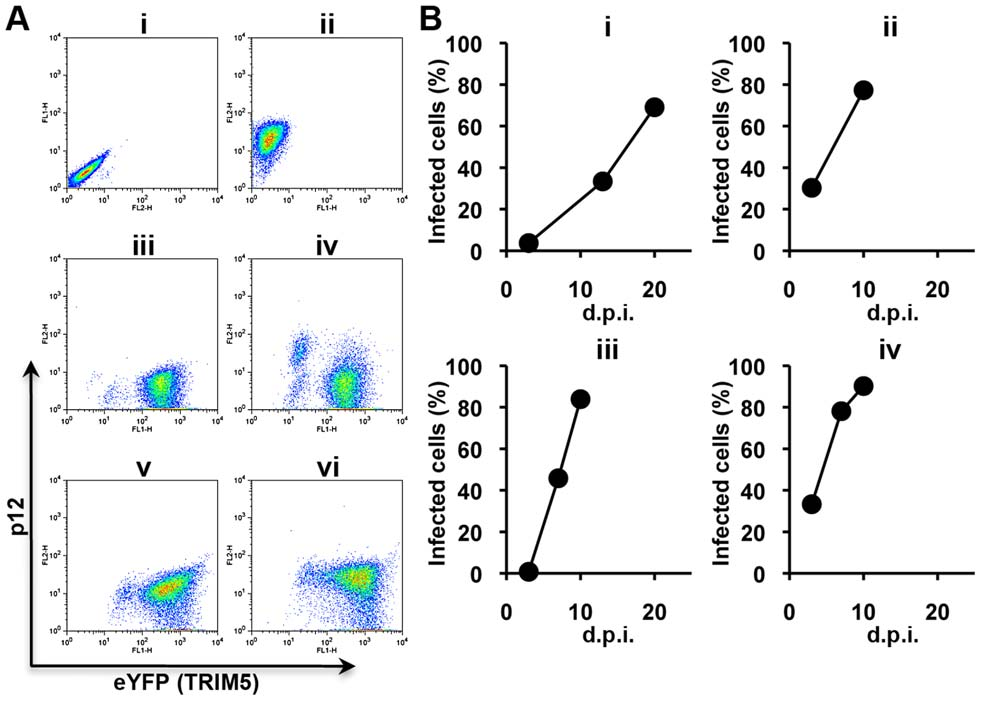
Selection of escape mutants from rhesus macaque TRIM5α. Escape mutants were derived by growing N-MLV on cell cultures containing mixed populations of TRIM5α-expressing and non-expressing MDTF cells. Virus spread was monitored by staining with an antibody specific to p12; the presence of TRIM5α was indicated by YFP. (A) Representative FACS profiles. (i) uninfected MDTF cells, (ii) MDTF cells 3 days post infection (d.p.i.) with N-MLV stained with anti-p12 (iii) – (vi) panels showing 90% TRIM5-positive cells at 0 d.p.i. (iii), 3 d.p.i. (iv), 13 d.p.i. (v) and 20 d.p.i. (vi) with N-MLV. Note that YFP-positive cell population shifted up into the dual-positive fraction. (B) Emergence of escape mutant in the culture. The percentage of p12 viral antigen positive cells in the culture was plotted against the time, d.p.i. Cells were passaged every 3 days for three weeks before cell-free virus passage to establish the next culture. The first and second cultures were performed in the mixed cell culture of TRIM5-positive cells with 10% TRIM5-negative cells, followed by the third and forth cultures with 100% TRIM5-positive cells. In the mixed cell cultures, p12-positive cell percentages were calculated for the YFP positive cells. Input RT activities: 1^st^ culture 514 mU; 2^nd^ culture 1151 mU; 3^rd^ culture 20 mU; 4^th^ culture 712 mU.

Low titres of N-MLV were used to infect a mixture of 90% TRIM5α-positive and 10% TRIM5α-negative cells. Virus spread was monitored by fluorescence staining using a monoclonal antibody directed against the viral p12 protein. As shown in Figure 2A, virus-infected cell populations were clearly distinguishable from virus-negative cells both in YFP-negative (TRIM5α-negative) and -positive (TRIM5α-positive) populations. Following infection of the first mixed culture, a small fraction of the YFP-positive cells appeared virus-positive as early as 3 d.p.i.; the virus spread into about 30% of the YFP-positive cell population within 13 d.p.i. and 60% by 18 d.p.i. (Figure 2B iv-vi). The culture supernatant was then collected, filtered, diluted tenfold and passed again in mixed cell culture for a further ten days (2^nd^ culture). To select mutant viruses, the filtered and diluted culture supernatant from the second mixed cell culture was inoculated onto 100% MDRh cells (the 3^rd^ and 4^th^ cultures). In contrast to wild type N-MLV, which at 1∶10 dilution had shown no virus expansion during the first 18 days of MDRh culture (Figure 1C), the virus from the second culture took 7 days to grow in 100% MDRh cells in the third culture (Figure 2B). This was not a result of different levels of viral input because virus with RT activity of 20 mU was inoculated in the third culture compared to 514 mU RT activity in the first inoculum. The virus at the end of the third culture was stored and referred to as rhesus TRIM5α-escape mutant 1 (REM1).

This procedure was repeated a further three times starting with N-MLV Stock A yielding REM2-4. In each experiment a similar time course was observed for isolation of a virus capable of unrestricted growth in the presence of rhTRIM5α. These results clearly demonstrate the utility of unrestricted refugia in the isolation of escape mutants from rhTRIM5α by allowing growth against a background of restriction.

The primary target for Fv1 and TRIM5α restriction is thought to be the viral CA protein. It was thus expected that escape mutations would map to the capsid so the nucleotide sequences of the capsid region of the viral *gag* gene was determined by direct sequencing of viral cDNA amplified from viral RNA in the culture supernatant. These data revealed that REM1 had acquired a single nucleotide substitution from T to G at nucleotide 1309 (AKV numbering) [37], which is predicted to produce a leucine to tryptophan substitution at amino acid position 10 in CA. The same L10W mutation in CA was seen in four independent experiments (isolates REM1-4) and no other mutations were detected in the capsid gene. This suggested that a small population of viruses carrying the L10W mutant may already exist in the virus stock prepared from the culture supernatant of chronically infected cells (Stock A) and the mutant was selected in the course of the virus passage experiments. As a first test of this hypothesis, the capsid gene of Stock A virus was amplified by RT-PCR and cloned. No sequence variation was observed in 50 independent clones (all 50 clones yielded sequences identical to N-MLV derived from pWN41) indicating that any minority species can only be present at very low levels.

We next derived a second stock of virus by transfection of 293T cells with self-ligated pWN41 provirus followed by three cell-free transfers to MDTF cells (Stock B). Two independent experiments with the new N-MLV stock following the same protocol used to isolate REM 1-4 resulted in the isolation of potential escape mutants REM5, 6. The only change in the capsid genes in these two viruses was a single T to A change at nucleotide 1627, giving rise to a V116D mutation. Four further experiments with freshly prepared Stock Bs gave rise to REM7-10; three carried single mutations resulting in L10W, H114N and H114R changes whereas REM10 carried three mutations causing L4S/A95D/S202G alterations.

A third set of experiments used Stock C, which was recovered directly following transfection of human 293T cells without passage through murine cells and should therefore have no mutations associated with reverse transcription (pWN41 encodes an ecotropic virus which will only infect murine cells), led to the isolation of REM11-15. Sequencing studies showed that these viruses carried mutations resulting in the following changes: G8D, N82D (twice), E92K and H114D.

**Figure 3:**
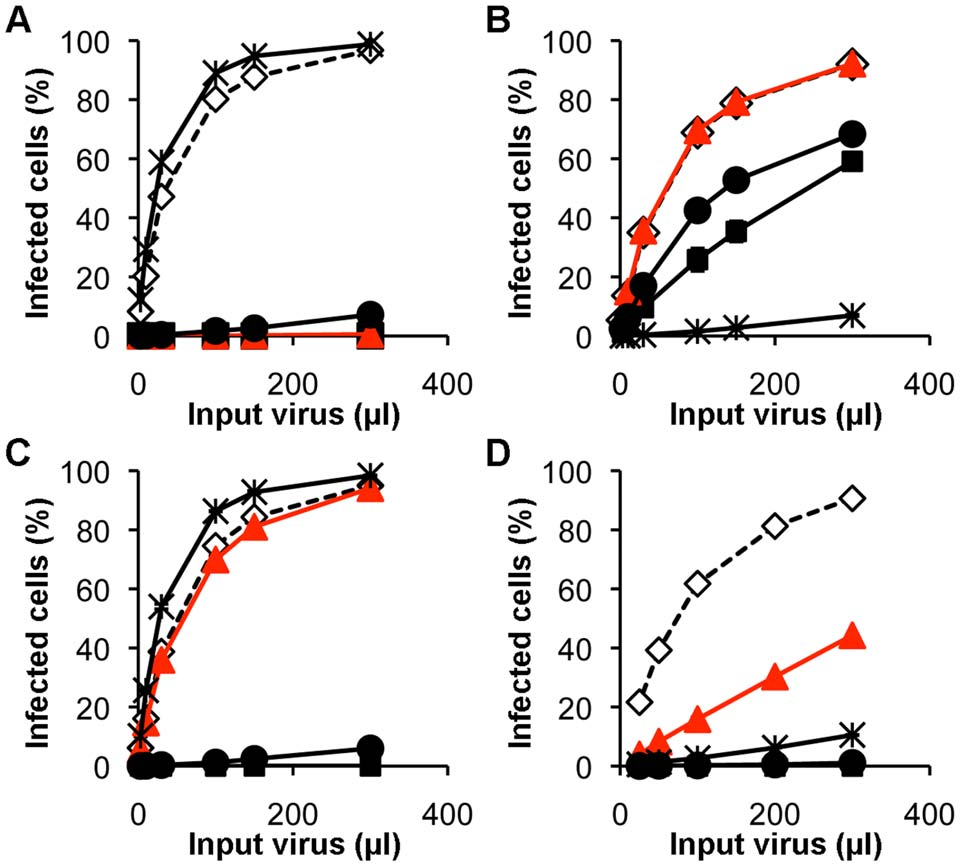
Restriction of N-MLV vectors carrying L10W and V116D. eGFP-encoding vector virus (A, wild type N-MLV; B, B-MLV; C, N-MLV(L10W); D, N-MLV(V116D)) were titrated on MDTF cells stably expressing restriction factors (TRIM5-negative, open diamond; human TRIM5α, filled square; rhesus TRIM5α, filled red triangle; Fv1^b^, filled circle; Fv1^n^, asterisk). Experiments were performed in triplicate; mean values from one representative experiment from three are plotted.

To confirm the importance of these mutations in escape from rhTRIM5α they were introduced by Quikchange PCR into the N-MLV vector, pCIG3N and the resulting viruses tested for growth with different restriction factors. On the basis of these analyses (Figure 3 and Supplementary Figure S1), the escape mutants could be placed into two groups depending on the degree of residual sensitivity to rhTRIM5α. One group, typified by L10W and comprising L10W, H114D, H114N, H114R and E92K showed equivalent titration curves in the presence and absence of rhesus TRIM5α (though E92K grew poorly compared to wild type N-MLV). The second group, typified by V116D, contained in addition G8D and N82D. This group showed slightly reduced infectivity in the presence of rhTRIM5α. Two of the single amino acid changes present in the triple mutant, L4S and A95D, gave rise to viruses that would fall in the second group but together show complete resistance. The S202G change appeared to have no effect on restriction, as might be expected for a residue in the (interior) C terminal domain of CA. Perhaps surprisingly, only one of the mutations that result in escape from rhTRIM5α, E92K, affects restriction by huTRIM5α. Furthermore, three of the mutations that give rise to resistance to rhTRIM5α, G8D, H114D and V116D confer sensitivity to Fv1^n^ while maintaining Fv1^b^ susceptibility. These data are summarized in Table 1.

**Table 1:** Summary of the restriction profiles of the escape mutants.

	Restriction			
Virus	HuTRIM5	RhTRIM5	Fv1b	Fv1n
wtN-MLV	++	++	++	−
B-MLV	+	−	+	++
L4S	++	+	++	−
G8D	++	+	++	++
L10W	++	−	++	−
N82D	++	+	++	−
E92K	−	−	−	−
A95D	++	+	++	−
H114D	++	−	++	++
H114N	++	−	++	−
H114R	++	−	++	−
V116D	++	+	++	++
L4S/A95D	++	−	++	−

For primary restriction data for these mutants, see Supplemental data (Figure S1). Similar results were obtained from three independent experiments.

++: more than 10-fold reduction of infectivity compare to wild type, +: 2- to 10-fold reduction of infectivity compare to wild type, -: less than 2-fold reduction of infectivity compare to wild type.

### Mutant capsids are resistant to rhesus macaque TRIM5α mediated disassembly

**Figure 4:**
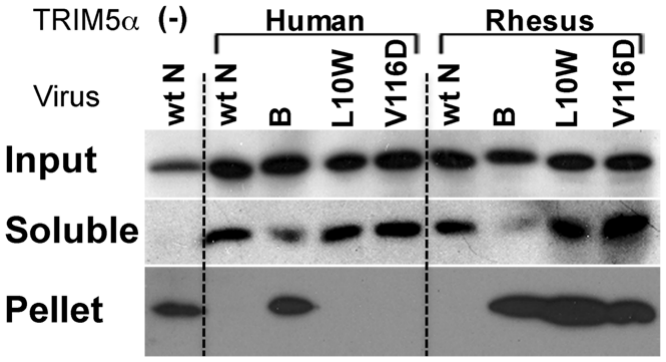
Capsid cores from escape mutants are not disassembled by rhesus TRIM5α. The interaction of TRIM5α with incoming capsid cores was studied in TRIM5α-expressing cells infected with VSV-G envelope-pseudotyped viruses. After lyzing infected cells in hypotonic buffer without detergent, cell extract was overlayered above 50% sucrose cushion and centrifuged. The presence of CA protein was examined in cell extract (upper panel; input), supernatant (middle panel; soluble) and pelleted (bottom panel; pellet) fractions. Representative results from one of three independent experiments are shown.

Since mutations at positions 10 and 116 have not previously been reported to affect interactions with Fv1 or TRIM5α we next set out to explore the properties of the L10W and V116D mutations. To examine the interaction between viral cores carrying the L10W and V116D mutations and rhTRIM5α, we tested whether such cores would resist rhTRIM5α mediated disassembly using the previously described fate-of-capsid assay that follows the premature capsid disassembly thought to occur in an infected cell following virus encountering TRIM5α [38]. As expected, wt N-MLV cores were pelleted in TRIM5α-negative cell extract (Figure 4, lane 1) and B-MLV capsid cores, but not wt N-MLV, L10W and V116D mutant capsid cores, were pelleted when huTRIM5α-expressing cells were infected (Figure 4, lanes 2–5). This result was consistent with the observations that huTRIM5α restricted wt N-MLV, L10W and V116D mutant viruses, but not B-MLV. In contrast, in the presence of rhTRIM5α, B-MLV and the L10W and V116D N-MLVs were pelleted, but wild type N-MLV was not detected in the pellet fraction (Figure 4, lanes 6–9). Therefore, L10W and V116D mutant viruses escape disassembly of capsid core caused by rhTRIM5α, an observation consistent with the lack of restriction of these mutant viruses by rhTRIM5α and emphasizing the species-specific nature of escape by L10W and V116D mutants from TRIM5α.

### Restriction profiles of L10 mutants and V116D by primate TRIM5αs

The selectivity difference of rhesus and human TRIM5αs prompted us to carry out a more detailed survey of the restriction profiles. Firstly, as only the L10W mutation was isolated through the *in vivo* passaging experiments we wondered what the effect of other residue types at position 10 in N-MLV CA might have on restriction factor sensitivity. To address this possibility, random amino acids were introduced at position 10 by PCR mutagenesis. Using this procedure fourteen of the possible nineteen substitutions and wt silent codon changes were recovered. The restriction data for rhTRIM5α are shown in Supplementary Figure S2 and summarized in Table 2. Out of the entire panel only two additional mutants, L10H and L10K, giving complete escape were identified. Two other substitutions (L10P and L10V) resulted in wt restriction phenotype; another gave non-infectious virus (L10R), whereas the remainder display intermediate phenotypes with restriction by TRIM5α at low virus titre but with only moderate restriction at higher virus titre. The variable phenotypes produced in this experiment highlight the importance of MLV residue 10 suggesting a direct involvement/interaction of this part of CA with rhTRIM5α in restriction of N-MLV but do not suggest a direct correlation between restriction activity and the size or charge of the amino acid residue at position 10.

**Table 2:** Restriction sensitivity of CA10 mutants.

	Amino acid at position 10
Full restriction	L, P and V
Partial restriction	A, C, D, F, N, Q, S and T
No restriction	H, K and W
Less infectivity	R

For primary restriction data from these mutants, see Supplemental data (Figure S2). Similar results were obtained from three independent experiments.

Next we tested the ability of a previously described panel of primate TRIM5αs to restrict the L10W, L10K, L10H and V116D mutant viruses [24]. Wild type N-MLV was restricted by all the ape and old world monkey TRIM5αs tested as well as some of the new world monkey TRIM5α including from Cotton-top tamarin and Brown capuchin (Table 3 and Supplementary Figure S3). All the L10 escape mutants were also restricted by ape TRIM5αs, which was perhaps not unexpected given the observation that huTRIM5α restricted the L10W mutant virus. However, among old world monkey TRIM5αs, only African green monkey TRIM5α restricted the L10 mutants and in each case these mutations also resulted in escape from restriction by the capuchin factor. On the other hand, the restriction profile of the V116D mutant virus was rather different. It was not restricted by orang-utan TRIM5α but was restricted by sooty mangabey and Brown capuchin TRIM5αs. These observations showed that whereas the viruses with mutations at position 10 showed a similar phenotype in terms of restriction by TRIM5α, the V116D mutant exhibited a different restriction profile by primate TRIM5α. These results imply that capsid recognition by TRIM5αs of different species may involve interactions with different amino acids on CA.

**Table 3:** Restriction sensitivity of L10 mutants and V116D to primate TRIM5αs.

	Restriction				
Origin of TRIM5	wt (10L)	L10W	L10H	L10K	V116D
Human	++	++	++	++	++
Gorilla	++	++	++	++	++
Orang-utan	++	++	++	++	−
Rhesus macaque	++	−	−	−	−
Sooty mangabey	++	−	−	−	++
African green monkey	++	++	++	++	++
Cotton-top tamarin	++	+	++	++	nd
Silvery marmoset	−	−	−	−	−
Squirrel monkey	−	−	−	−	nd
Brown capuchin	++	−	−	−	++

For the complete profile of titration curves, see Supplemental Data (Figure S3). Representative results were shown from at least three independent experiments.

++; restricted more than 10-fold compared to wild type, -; restricted less than two-fold compared to wild type, and: +; partially restricted in between above two criteria.

Chimeric TRIM5α of human RBCC domain fused with specified B30.2 domain nd, not done.

### Involvement of B30.2 V1 and V2 regions in N-MLV CA recognition

**Figure 5:**
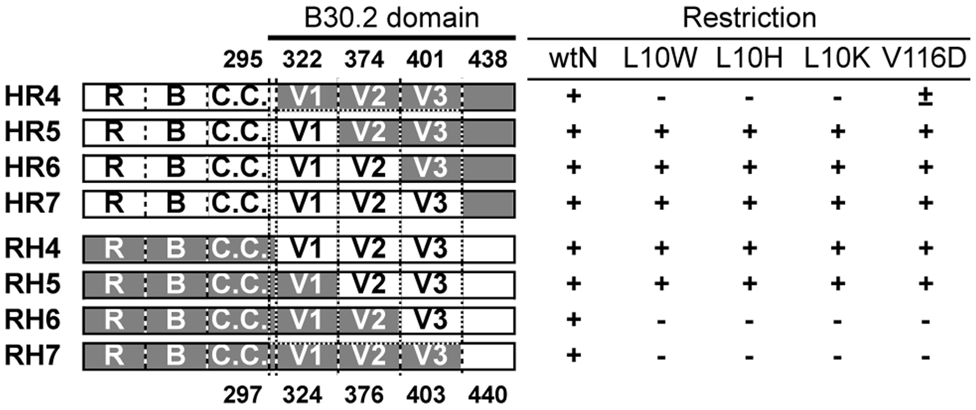
Mapping regions of the B30.2 domain involved in MLV restriction. The left half of the figure shows schematic views of previously described [16] chimeras between human (white shading) and rhesus macaque (gray shading) TRIM5α. The right side summarizes restriction data of mutant viruses by these TRIM5α. For a full panel of titration curves, see Supplemental Data (Figure S4). Representative results are shown from one of three independent experiments. MDTF cells were transduced with chimeric TRIM5α and cultivated for more than 4 weeks until cells reached the steady expression level of TRIM5α. YFP expression was examined 4 weeks after transduction and more than 95% of total cells expressed YFP. TRIM5α-expressing cells were infected with mutant viruses and the ratio of infected to non-infected cells was calculated in the YFP-positive cell fraction. +; restricted more than 10-fold compare to wild type, −; restricted less than two-fold compare to wild type, and ±; partially restricted.

These species differences in restriction profiles of the escape mutants allowed us to use a previously described series of chimeric human/macaque TRIM5αs [16], to ask which of the V1, V2 and V3 variable regions of the TRIM5α B30.2 domain are important for CA binding (Figure 5, Supplementary Figure S4). Each chimera was designed to contain a different combination of the variable regions of the B30.2 domain (Figure 5). Both human and rhesus TRIM5α restrict N-MLV and so not surprisingly MDTF cells stably expressing each of the eight chimeric TRIM5αs restricted wild type N-MLV. The position 10 mutants (L10W, L10K and L10H) show identical patterns of sensitivity to the chimeric constructs, being unrestricted by HR4, RH3 and RH4, but restricted by the other chimeric TRIM5α, suggesting that when either the V1 or V2 region is derived from huTRIM5α, the mutants are restricted, whereas when both V1 and V2 regions were derived from rhTRIM5α, the mutant N-MLV was not restricted. The V116D escape mutant also displays an identical profile to that of the position 10 mutants, supporting the idea that both V1 and V2 regions are involved in TRIM5α-mediated restriction of MLV.

### The effect of β1-β2 mutations on HIV-1 susceptibility to Rhesus TRIM5α

The N-terminal domain of HIV-1 and MLV CAs share very similar structures [32], including the β-hairpin region. It therefore seemed possible that this region might affect sensitivity of HIV-1 to rhesus TRIM5α. To address this question, each amino acid between position 5 and 12 on HIV-1 capsid, which form the N-terminal β-hairpin loop, was substituted with a lysine residue. In addition, the methionine residue at position 10 of HIV-1 capsid was substituted randomly by mutagenesis PCR. However, although several of these mutations severely impaired Gag function, none of the viable HIV-1 mutants examined in this study, escaped rhTRIM5α (Supplementary Figure S5), implying that unlike MLV, the N-terminal β-hairpin loop of HIV-1 capsid does not seem to affect the restriction activity of TRIM5α. Based on these results, the ways by which rhTRIM5α recognizes viral capsid seems to be different between HIV and MLV.

### Structure of N-MLV CA carrying the L10W mutation

On inspection of the structure of the MLV CA-NtD (Mortuza et al., 2004) it was apparent that although the side chains of both residues 10 and 116 were surface exposed on an assembled capsid structure, neither lay in the α4–α6 region, previously proposed as the Fv1 binding site (Mortuza et al., 2008). Residue 116 was located on the second turn of α6 on the face opposite the α4–α6 region while residue 10 was even more remote, located on the N-terminal β1–β2 hairpin. To further examine how the L10W mutation might effect rhTRIM5α restriction of MLV we determined the crystal structure of the CA-NtD of this variant at 2.0Å resolution. The structure has been refined to a final R_work_/R_free_ of 17.4%/24.4% respectively and has excellent geometry with only 3 residues in the additionally allowed region of the Ramachandran plot (See Table 4 for details of crystal parameters, phasing and data refinement statistics).

**Table 4:** Statistics of data collection, phasing and refinement.

	Ncap-L10W
**Data collection**	Home-Source
Space group	C2
Cell dimensions a, b, c (Å), α, β, γ (°)	71.9, 33.6, 59.3, 90, 111.9, 90
Wavelength (Å)	1.5418
Unique reflections	9015
Resolution range (Å)	25-2.0 (2.07-2.0)
Rsym (%)	4.4 (18.5)
I/σ(I)	25.1 (6.29)
Completeness (%)	99.6 (97.0)
Redundancy	3.4 (2.9)
**Phasing (MR Phaser)**	
Z-score	12.3
LLG	590
**Refinement**	
Resolution (Å)	25-2.0 (2.29-2.0)
R_work_/R_free_ (%)	17.4/24.4 (18.0/26.6)
*No atoms (residues)*	
Protein	1088 (135)
Water	99
Glycerol	6 (1)
*B-factors (Å^2^)*	
Wilson	30.3
Protein	35.8
Glycerol	47.7
Water	41.8
Overall	36.3
*R.M.S. deviations*	
Bond lengths (Å)	0.009
Bond angles (°)	0.977

**Figure 6:**
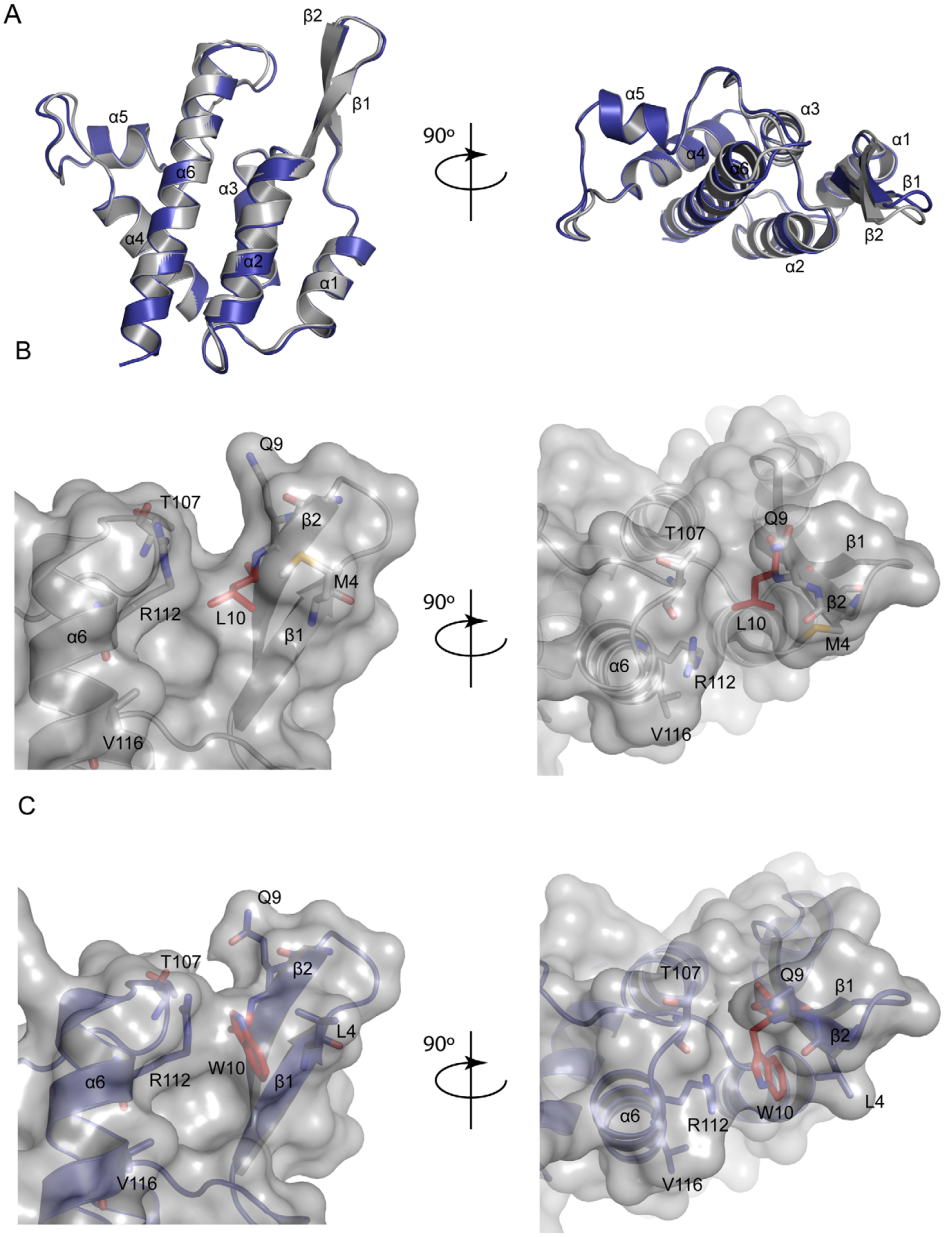
Structure of MLV CA-NtD (L10W). **A**) Structural superimposition a monomer of the CA-NtD from N-MLV (grey) and that of N-MLV(L10W) (blue), side (upper) and top (lower) views are shown. Structures are displayed in cartoon representation, secondary structure elements are labeled. **B**) Groove between β1–2 and α5–α6 in wt N-MLV. L10 in the base of the groove is shown in red. R112, T107, and Q9 lining the grove are shown as sticks. A transparent molecular surface is shown over the molecule same orientation as **A**. **C**) W10 and R112 block the groove in the structure of N-MLV L10W. The position of V116 on α6 is also shown.

The structure of L10W was typical of a retroviral CA-NtD consisting of an N-terminal β-hairpin and a core bundle of six α-helices. A comparison with the native MLV CA-NtD structure (Figure 6A) revealed an essentially identical backbone conformation. Pairwise comparisons of L10W with wt N-MLV CA-NtD gave an RMSD of 0.6Å across equivalent Cα atoms. However, minor deviations were seen at the top of helix-6 and in the loops and side chains on the surface of the molecule. In particular, the L10W mutation was clearly visible in the electron density (Supplementary Figure S6) and was located on the inward facing side of strand β2 adjacent to the α5-α6 loop and the top of helix α6.

Examination of the wt N-MLV capsid surface in the proximity of L10 (Figure 6B) reveals the presence of a shallow channel between the β1–β2 hairpin and residues at the top of α6 and in the α5–α6 loop. The channel walls are formed by side-chains of M4 and Q9 from the β1–β2 hairpin together with R112 and T107 from the α5–6 loop. At the bottom of the channel the aliphatic L10 side-chain forms the base. However, in the L10W structure (Figure 6C) the much bulkier tryptophan side-chain protrudes into the channel along with R112 guanidinium moiety. Together they adopt a near planar arrangement reminiscent of a similar interaction seen in the structure of PSIV capsid [39]. Significantly, this side-chain configuration in L10W reduces the depth of the (β1–β2)-α6 channel by some 2.3Å, effectively closing the cavity completely. To test the importance of the W10 – R112 interaction for restriction, the R112 amino acid was mutated to an alanine in presence of both L10 and W10. Both viruses were susceptible to restriction by rhTRIM5α (Supplementary Figure S7). The observed structural change thus provided a simple potential explanation for the rhTRIM5α escape phenotype suggesting that in wt N-MLV the (β1–β2)-α6 channel constituted at least part of the rhTRIM5α binding site and that by blocking this channel in L10W the interaction with rhTRIM5α was abolished or sufficiently diminished to allow the virus to evade restriction.

## Discussion

To better understand the interaction between TRIM5α and its viral target, we set out to isolate a panel of novel N-MLV escape mutants. Noting that direct inoculation of cells expressing a given restriction factor led, depending on the initial virus titre, either to rapid break-through of the block in replication or the absence of replication (Figure 1), we devised a procedure allowing replication against a background of restriction. We reasoned that the infection of a mixture of restricting and non-restricting cells would allow both multiple rounds of unrestricted replication as well as selection for viruses that were unrecognized by restriction factor. Experiments of the kind shown in Figure 2 illustrate the utility of such an approach and have resulted in the isolation of a number of MLV mutants that escape restriction by rhTRIM5α, apparently by escaping premature capsid disassembly (Figure 4). The lack of variation in mutants obtained with Stock A compared with that from Stocks B and C points to the possible accumulation of variant viruses in multi-passaged virus stocks even in the absence of overt selection or apparent sequence variation and emphasize the need for using freshly recovered virus in such studies.

### β-hairpin mutants extend the MLV CA-restriction factor interface

**Figure 7:**
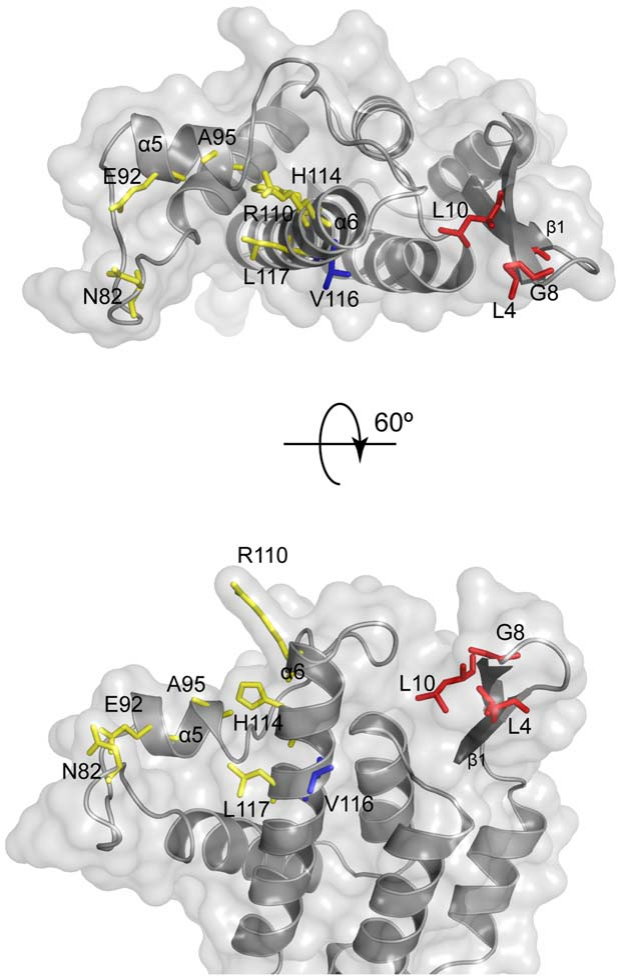
Location of MLV restriction determinants. Determinants of MLV restriction, side (lower) and top (upper) views of the N-MLV CA-NtD structure are displayed. The protein backbone is shown in cartoon representation together with the semi-transparent molecular surface. Residues involved in restriction factor specificity are shown as sticks. Determinants of N-B tropism are shown in yellow. Leu 10 that when substituted by Trp, Lys or His confers resistance to rhTRIM5α is shown in red. V116 that when substituted by Asp confers resistance to rhTRIM5α and gives rise to Fv1n sensitivity is shown in blue.

The mutations identified by these passaging experiments together with *in vitro* mutagenesis studies all lie on the outer “top” surface of MLV CA (Figure 7) and can be divided into two groups. One group (Class-I) comprises N82D, E92K, A95D, and H114D/R/N, mutations that are within the previously defined Fv1 binding pocket in the loop region between helices 4 and 6 of CA [27], [30]. These mutations result in escape from rhesus TRIM5α mutations at these positions can affect susceptibility to Fv1. The other escape mutants, L4S, G8D, L10W, L10K, L10H and V116D are the founder members of a new group (Class-II) located on the opposing side of the central α6 helix and in the **β**1–**β**2 N-terminal **β**-hairpin. Residue 110 (R in N-MLV and E in B-MLV) is located on the top of α6 and is often referred to as the major specificity determinant of restriction as it was the first residue shown to determine susceptibility to Fv1 or TRIM5α, [8], [19], [31]. Our data now add further weight to the significance of residue 110 revealing that it is located at the centre of an extended restriction factor binding-site. This site encompasses the entire CA top surface from N82 across the region containing Class-I mutants to residues 8 and 10 in the **β**-hairpin. The G8D and mutations at L10 are of particular interest as they are located in a region of CA, the N-terminal **β**1–**β**2 hairpin, where mutations affecting the interaction of N-MLV with TRIM5α or Fv1 have never before been reported. The structure and properties of the L10W mutant (Figure 6) and the effect of the R112A change (Supplementary Figure S7) strongly suggest that the (**β**1–**β**2)-α6 channel present on CA forms part of the rhTRIM5α binding site. The observation that G8D and V116D cause susceptibility to Fv1^n^ further suggests that the previous defined Fv1 binding pocket must be extended to include this region.

It is noteworthy that residues 10 and 82 are 29 Å (Cα-Cα) apart on either side of the exposed surface of CA monomers making up the core [32]. Moreover restriction sensitivity is affected by a number of amino acids (e.g. 92, 95, 110, 114, 116, 117) that are distributed between these two positions, covering much of the viral core that is exposed to cellular factors after entry into the cytoplasm of newly infected cells (Figure 7). Many of these residues posses poorly ordered side chains so their contribution to αrestriction factor binding seems most likely through side chain charge-charge or hydrophobic interactions at the molecular interface. Indeed many of the escape mutations we have isolated involve introduction or loss of charged amino-acids. Further, in cases of mutations affecting tropism for which we have detailed structural information, R110E [27] and L10W (this paper) there is no evidence for major change in the overall conformation of CA, indicating that mutations in the N terminal **β**-hairpin loop are not affecting the structure of other regions of CA for example the α4–α6 region. Taken together, these observations are consistent with the involvement of much, if not all, of the surface of N-MLV CA in recognition and binding by rhesus TRIM5α. Certainly the surfaces of the two proteins appear to lie in sufficiently close proximity that the introduction of charged residues in a variety of different positions prevents binding. An extensive recognition surface fits well with distance measurements of the B30.2 domain from TRIMs closely related to TRIM5 [40], [41] that reveal that the specificity determiningV1 and V3 regions are separated by about 30 Å, a number very similar to that between the two sides of CA residues (see above). However, attempts to identify regions of TRIM5α that bind specific CA residues using TRIM5α chimeras such as those illustrated in Figure 5 have not met with great success; rather it appears that well separated TRIM5α regions act in combinatorial fashion to determine restriction specificity.

### Comparison of the restriction factor binding sites on different retroviruses

TRIM5α is capable of recognizing at least four genera of retroviruses despite extensive differences in primary sequences of CA [23], [24], [25]. However CA's show extensive structural conservation particularly in the core fold of helices 1–4 [42]. It thus appears likely that it is some shared feature of overall structure that represents the target for TRIM5α rather than direct sequence specific recognition alone. Loop exchange studies involving different lentiviruses suggest that the primary target for the restriction factor lies in the cyclophilin A binding loop, in a region analogous to the originally defined Fv1 binding site of MLV [33], [34]. The importance of this region is emphasized by the recent isolation of an HIV-1 escape mutant from rhTRIM5α, V86M, by methods similar to those used here [43] and the roles of HIV-2 P120 and G116 in resistance to cynomolgus macaque TRIM5α [35], [44]. HIV-1 V86 and MLV N82 both lie just C-terminal to helix 4 while HIV-1 G116 and HIV-2 P120 lie close to position 110 of MLV. Further, domain swap experiments also suggest a role for the **β**-hairpin region of SIV CA protein in the evasion from rhTRIM5α-mediated restriction [36].

Based on these results and our success of producing and mapping **β**-hairpin escape mutants of N-MLV that could evade restriction by rhTRIM5α, it seemed logical to extend these studies and test for commonality of binding by examining the restriction phenotypes of **β**-hairpin mutants in HIV-1. Moreover, a comparison of the CA-NtDs revealed that, although wider, the **β1**-hairpin-α6 cleft is also present in HIV-1 (Supplementary Figure S8). Therefore, it might be expected that the introduction of bulky or charged side chains into this channel would alter HIV susceptibility to TRIM5α in the same way as in N-MLV. To this end, the studies presented in supplementary Figure S5 were undertaken. However they showed that viable single amino acid mutations in the HIV-1 **β**-hairpin had no effect on restriction by rhTRIM5α. These data suggest that the **β**-hairpin-α6 cleft is not a shared target for all viruses, implying that an as yet unidentified binding region near the **β**-hairpin remains to be discovered. In any event, it seems most likely that lentiviruses have an extended TRIM5α binding site in analogous to that seen in MLV.

### Human and rhesus TRIM5α restriction of MLV, the devil is in the detail

The results of the initial passaging studies (Figure 1) together with phenotypic analysis of our escape mutants (Figure 5) suggest that there are significant differences in the recognition of N-MLV by rhesus and human TRIM5α. From the outset, restriction of N-MLV by huTRIM5α was much more potent to the extent that we have been unable to overcome N-MLV restriction by the hu-TRIM5α. Moreover, it is also refractory to all but one of the changes isolated that allow escape from the rh-TRIM5α (Table 1). The one exception, E92K, was resistant to all restriction factors tested but showed a significant reduction in titre. These observations suggested that the interaction of huTRIM5α with N-MLV was either stronger and can elicit a more powerful host cell response or that the CA-TRIM5α interface is different and does not include the residues in the **β**-hairpin-α6 channel. These observations are somewhat perplexing given the degree of similarity of the TRIM5α B30.2 domains. However, it is notable that L10 and V116D mutations have differential effects on restriction by TRIM5αs derived from other species. For example V116D shows no restriction by orang-utan TRIM5α but is restricted by sooty mangeby whereas L10W shows the converse sensitivity (Table 1). Further, of the fourteen TRIM5αs we have previously examined only two, pigtailed macaque and sooty mangabey, show identical restriction profiles against a panel of ten different viruses [23], [24], [25].

Taken together, these variable determinants of restriction imply that TRIM5α recognizes its targets in a manner significantly different from many protein-protein interactions that involve a small number of highly conserved residues, exemplified by the interaction betweens HIV-1 CA and the cellular protein cyclophilin A [45]. Rather the interactions are predominately weak and spread over a large surface. Avidity could then be provided by multivalent binding to the regularly spaced CA molecules assembled on the viral core [26], [27], [28]. This notion is further supported by recent structural data suggesting that formation of a complementary TRIM5α lattice is actually a requirement for a productive interaction of the restriction factor with capsid αassemblies (Ganser-Pornillos et al, 2011). With the idea of lattice complementarity in mind it is also interesting to note that although differences in conformation around the top of Helix-6 between wt and L10W are only very small (∼1.2Å) this region is centrally located in the extended restriction factor binding-site. It is plausible that such small changes whilst not affecting hexamer or CA lattice formation when propagated throughout an entire capsid lattice could profoundly affect any lattice complementarity, altering restriction factor susceptibility and resulting in viral escape from some TRIM5αs. Whatever the case, retroviral pathogens comprise a wide range of sequence divergent but structurally related assembled repeated capsid moieties [42]. Perhaps weak interactions spread over a large binding surface are implicit in the design of a defence mechanism intended to recognize these diverse targets.

## Materials and Methods

### Cells

Tail fibroblast cells from *Mus dunni* (MDTF), MDTF cells that stably express restriction factors, and human 293T cells were maintained in Dulbecco's modified Eagle's medium (DMEM) supplemented with 10% fetal calf serum and antibiotics. MDTF cells expressing human and rhesus macaque TRIM5α (MDHu and MDRh) were established by an end-point dilution method. Stable, unselected expression of TRIM5α was observed in transduced cells for at least one month after cloning.

### DNA

The cloning of human TRIM5α from TE671 and a chimeric human/rhesus macaque TRIM5α (human RBCC domain fused with rhesus macaque B30.2 domain) have been described previously [8], [24]. This chimeric human/rhesus macaque TRIM5α was used throughout this study to generate and select escape mutants of N-MLV, thereby allowing a direct comparison of interactions with the human and rhesus macaque B30.2 domains, avoiding confounding effects of the coiled coil region [16], [46], [47], and will be referred to as rhesus TRIM5α. Generation of transduction vectors expressing TRIM5α and Fv1 using the Gateway system was also described previously [24], [48]. Single nucleotide mutations were introduced into the capsid genes of Gag-Pol vector plasmids (pCIG3N and pCIG3B) and N-MLV provirus plasmid (pWN41 [17]) by the QuickChange mutagenesis PCR protocol. Random mutations were introduced at position 10 on MLV capsid by the QuickChange mutagenesis PCR using the following primer pair: forward 5′- ggggggtaatggtcagNN(G/T)cagtactggccgttttc -3′ and reverse 5′- gaaaacggccagtactg(C/A)NNctgaccattacccccc -3′, where N indicates any nucleotide and G/T and C/A indicate G or T and C or A, respectively. The sequences of the other primers used to prepare mutants are available upon request. All introduced mutations were verified by DNA sequencing.

### Viruses

Single-cycle replication viruses were produced by transfection of VSV-G envelope (pcz VSV-G), retroviral gag-pol (pCIG3N and pCIG3B for N- and B-tropic viruses, respectively) and viral genomic plasmids (pczCFG2fEGFPf) into 293T cells by a conventional calcium phosphate method as described previously [48] or using Turbofect (Fermentas). Sixteen hours after transfection, cells were treated with 10 mM sodium butyrate for 6 hours, followed by replacement of the culture supernatant with fresh growth medium. Virus-containing supernatant was harvested 48 hours after transfection, filtered through a 0.45 µm pore filter disk and stored at −80°C until usage. Three stocks of replication competent N-MLV were used. One (Stock A) was prepared from the culture supernatant of MDTF cells transfected with N-MLV provirus (pWN41) followed by multiple (>20x) passage of the transfected cells. The second (Stock B) was generated by transfection of 293T cells with pWN41, transfer of the filtered supernatant to MDTF cells followed by three cell passages. The third (Stock C) was unpassaged supernatant of 293T cells transfected with pWN41. For transfection, pWN41 DNA was digested with Hind III to separate the permuted provirus from the vector, and purified viral DNA fragments were self-ligated with T4 DNA ligase at 16°C overnight. After purification, the ligated DNA was introduced into MDTF/293T cells using a commercial transfection reagent according to the manufacturer's instructions (TurboFect, Fermentas). Transfected cells were re-fed with fresh medium after 16 hours; culture supernatants were harvested after 48 hours.

Virus production in newly transfected/infected cells was monitored by protein blotting of pelleted culture supernatants with monoclonal antibodies specific to p12 and CA viral antigens (prepared from the culture supernatant of hybridomas 548 and R187 [ATCC]) or with a RT assay kit used according to the manufacturer's instructions (C-type RT assay kit, Cavidi Tech, Sweden).

#### Mutant virus selection

Target cells (MDTFs or rhTRIM5α expressing MDTFs or specified mixtures of the two cell types) were seeded on a 6-well plate at 2×10^5^ cells per well one day before virus inoculation. Replication competent N-MLV was added to target cells at a titer equivalent to 514 mU of RT activity, followed by washing cells three times with the growth medium 16 hours after infection. The input virus was allowed to grow in the culture by passing infected cells and virus spread was assessed by fluorescent staining. Once the majority of restriction factor positive cells were infected with N-MLV, the culture supernatant was harvested, filtered, and then stored at −80°C until usage. The filtered supernatant was diluted 1∶10 with growth medium and inoculated onto fresh, restriction factor-expressing cells. This virus passage cycle was repeated several times until the virus spread rapidly in restriction factor positive cells.

### FACS

Virus spread in infected cells was monitored by detection of an intracellular virus antigen, p12. Infected cells were harvested by trypsinization and washed with PBS, followed by fixation with 1.25% ice-cold paraformaldehyde in PBS. All of the following procedures were performed on ice. Fixed cells were washed once with permeabilization buffer (0.5 mg/ml digitonin and 1% bovine serum albumin [BSA] in PBS), followed by incubation with anti-p12 monoclonal antibody diluted in permeabilisation buffer. After washing three times with wash buffer (5% Tween-20 and 1% BSA in PBS), cells were then stained with anti-mouse IgG conjugated with AlexaFluor 594 (Invitrogen) diluted in permeabilisation buffer. After washing three times, cells were re-suspended in 1.25% paraformaldehyde in PBS and analysed by FACSCalibur within four hours.

Single-cycle replication experiments were performed as previously described [24], [48] using a delivery vector encoding restriction factor and eYFP and a tester virus encoding eGFP. Two colour FACS was carried out with a BD LSR II machine.

### Sequencing of the capsid gene

Virus containing culture supernatant was harvested and filtered through a 0.45 µm-pore filter disk. The filtered supernatant was stored at −80°C until usage. The supernatant was treated with MgCl_2_ and RNase-free DNase I at 37°C for 2 hours to degrade contaminating DNA. DNase I-treated supernatant was subjected to viral RNA extraction using a commercial kit (Viral RNA mini kit, QIAGEN). A single strand cDNA spanning the capsid region was synthesized from viral RNA as a template with a primer designed in the protease gene (5′-gggttatcctgggttcaggggggggctcctgaccctg-3′) using a commercial kit (Transcriptor High Fidelity cDNA synthesis kit, Roche). The cDNA synthesized was used as a template to amplify viral capsid gene with a primer pair designed in viral matrix (5′-gtctcgcctgcggggcaaaagag-3′) and protease (the same as above) genes using PfuUltra high fidelity DNA polymerase (Stratagene). The PCR product was gel purified, and nucleotide sequence was determined by direct sequencing.

### Fate-of-capsid assay

The fate-of-capsid assay was performed as previously described [38] with minor modifications. MDTF cells expressing TRIM5α (5×10^6^ cells in a 80 cm^2^ tissue culture flask) were pre-plated one day before assay. Cells were inoculated with freshly prepared, chilled virus (14 ml per flask with 5 ug/ml polybrene) from the filtered culture supernatant of transiently transfected 293T cells. After incubation on ice for 30 minutes, cells were incubated at 37°C for 4 hours. Cells were then washed three times with ice-cold PBS and treated with 1 ml of 7 mg/ml pronase at room temperature for 5 minutes. Cells were re-suspended in ice-cold growth medium and washed three times with ice-cold PBS. Cells were re-suspended in 2.5 ml of hypotonic lysis buffer [10 mM Tris-HCl (pH 8.0), 10 mM KCl, 1 mM EDTA and protease inhibitor cocktail] and incubated on ice for 15 minutes. Swollen cells were lysed in a 7 ml-Dounce homogenizer with a ‘tight’ pestle (15 gentle strokes) and cell lysates cleared by centrifugation at 4°C and 1,850 xg for 3 minutes. Cleared cell extracts (2 ml) were layered over 50% sucrose cushions prepared in PBS and centrifuged at 4°C and 125,000 xg for 2 hours in a Beckman SW41 rotor and 100 µl of the cell extract was collected as a ‘input’ fraction. After the centrifugation, 100 µl of the topmost portion of the supernatant was collected as a ‘soluble’ fraction. The pellet was re-suspended in 100 µl 1×SDS sample loading buffer (‘pellet’ fraction). Samples were separated on acrylamide gels and blotted for CA as described above.

### Protein crystallisation and structure determination

The CA N-terminal domain of N-MLV(L10W) was crystallised by sitting-drop vapour diffusion using a reservoir solution containing 24% PEG-8000, 0.1M phosphate citrate, pH 5.0 mixed with an equal volume of protein solution (14 mg/ml). Prior to data collection, crystals were transferred into a cryoprotectant of reservoir solution containing 20% glycerol and flash frozen in liquid nitrogen. X-ray diffraction data were collected at 100° K on a Rigaku Micromax-007HF with a Raxis-IV detector to a resolution of 2.0Å and processed using the HKL program package [49]. Crystals belonged to the space group C2 with the cell dimensions a = 71.9Å, b = 33.6Å, c = 59.3Å, β = 111.9°.

The structure of N-MLV(L10W) was determined by molecular replacement using a monomer of N-MLV CA-NtD (1U7K) as a search model in PHASER [50]. A single molecule was present in the asymmetric unit (Z-score 12.3, LLG 590). The model was rebuilt using Arp/Warp (Morris et al., 2003) followed by iterative rounds of refinement and model building in PHENIX [51] and COOT [52]. Nine TLS groups were included in final round of refinement as determined by TLSMD [53]. The structure was refined to a final R_work_/R_free_ of 17.4%/24.4% respectively.

### Coordinates

The coordinates and structure factors of N-MLV(L10W) have been deposited in the Protein Data Bank under accession number 2y4z.

## Supporting Information

Figure S1 Restriction of N-MLV vectors carrying various escape mutations. eGFP-encoding vector virus (A, H114D; B, H114N; C, H114R; D, L4S; E, A95D; F, L4S/A95D; G, L4S/S202G; H, A95D, S202G; I, S202G; J, G8D; K, N82D; L, 92K) were titrated on MDTF cells stably expressing restriction factors (TRIM5-negative, open diamond; human TRIM5α, filled square; rhesus TRIM5α, filled red triangle; Fv1^b^, filled circle; Fv1^n^, asterisk). Experiments were performed in triplicate; mean values from one representative experiment from three are plotted.
(TIF)

Figure S2 Restriction by rhesus macaque TRIM5α of N-MLV carrying different amino acids at CA position 10. A variety of amino acids were introduced at position 10 of CA by random mutagenesis. Unique isolates were tested for growth in the presence (solid line and filled squares) or absence (dotted line and open diamonds) of rhesus macaque TRIM5α. One representative result from three independent experiments is shown. These data are summarized in Table 2.
(TIF)

Figure S3 Titration curves of selected N-MLV escape mutants in the presence of various primate TRIM5α. Chimeric TRIM5α constructs expressing the human RBCC domain fused with different primate B30.2 domains [24] were used to test restriction of wild type and L10W, L10H, L10K and V116D escape mutant virus. Titration curves in the presence of ape, old world monkey and new world money TRIM5α are shown in green (left column), red (middle column) and blue (right column), respectively. One representative result is shown from three independent experiments. These data are summarized in Table 3.
(TIF)

Figure S4 Titration curves of N-MLV escape mutants in the presence of chimeric TRIM5α. Titration curves in the presence of human-rhesus TRIM5α chimeras are shown in the left column and those in the presence of rhesus-human TRIM5α chimera are shown in the right column. One representative result from three independent experiments is shown. These data are summarized in Figure 5.
(TIF)

Figure S5 Titration curves of HIV-1 with lysine-substitutions within the CA β-hairpin loop. Lysine substitutions were introduced at amino acid positions 5 through 12 of HIV-1 CA as well as random amino acid mutations at position 10. These were tested for growth in the presence (solid line and filled squares) or absence (dotted line and open diamonds) of rhTRIM5α. Data for the I6K, G8K and H12K mutants showed much reduced infectivity and are not shown. One representative result from two independent experiments is shown.
(TIF)

Figure S6 Stereo 2Fo-Fc electron density. Maps contoured at 1.0σ around the region of N-MLV containing either L10 (1U7K) in panel A or W10 (2Y4Z) in panel B are shown. The protein main-chain is shown in cartoon representation, residue side chains are shown in stick representation, L10 and W10 are highlighted in red.
(TIF)

Figure S7 Mutation R112A abolishes relief of TRIM 5α restriction by L10W. Single cycle experiments to examine the effect of the R112A mutation on restriction of wt and L10W N-MLV by TRIM5α. Infection was tested in the presence (filled squares) or absence (open diamonds) of rhTRIM5α. Panel A, N-MLV carrying the L10W mutation; Panel B. N-MLV with R112A; Panel C, N-MLV with L10W and R112A. One representative result from three independent experiments is shown.
(TIF)

Figure S8 The β1-β2-α6 cleft in HIV-1 CA-NtD. Side (lower) and top (upper) views of the HIV-1 CA-NtD structure are displayed (PDB ID 1M9C). The protein backbone is shown in cartoon representation together with the semi-transparent molecular surface. Residues in the amino-terminal **β**-hairpin that were mutated in order to look for escape from rhesus TRIM5α restriction are shown in stick representation. The cleft between **β**1-**β**2 and helix 5′ at the top of a6 is much wider in HIV than in MLV; *cf*. Figure 6.
(TIF)
