# Supplementary figures and images for: Novel Escape Mutants Suggest an Extensive TRIM5α Binding Site Spanning the Entire Outer Surface of the Murine Leukemia Virus Capsid Protein
Source: PLoS Pathog. 2011 Mar 31;7(3):e1002011. doi: 10.1371/journal.ppat.1002011 (PMC3068999; doi:10.1371/journal.ppat.1002011)

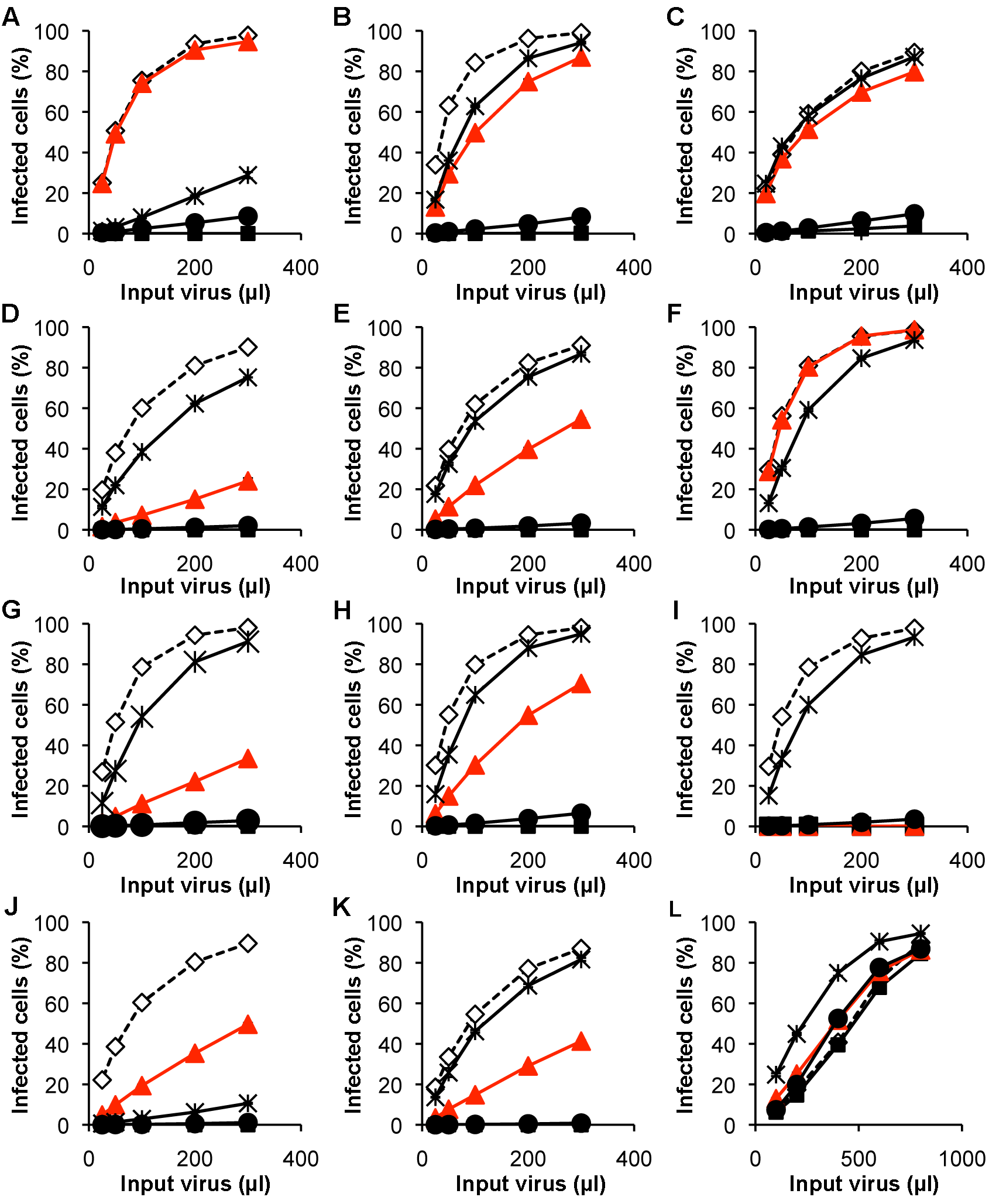

Supplement: Figure S1 — Restriction of N-MLV vectors carrying various escape mutations. eGFP-encoding vector virus (A, H114D; B, H114N; C, H114R; D, L4S; E, A95D; F, L4S/A95D; G, L4S/S202G; H, A95D, S202G; I, S202G; J, G8D; K, N82D; L, 92K) were titrated on MDTF cells stably expressing restriction factors (TRIM5-negative, open diamond; human TRIM5α, filled square; rhesus TRIM5α, filled red triangle; Fv1b, filled circle; Fv1n, asterisk). Experiments were performed in triplicate; mean values from one representative experiment from three are plotted. (TIF) [file ppat.1002011.s001.tif]

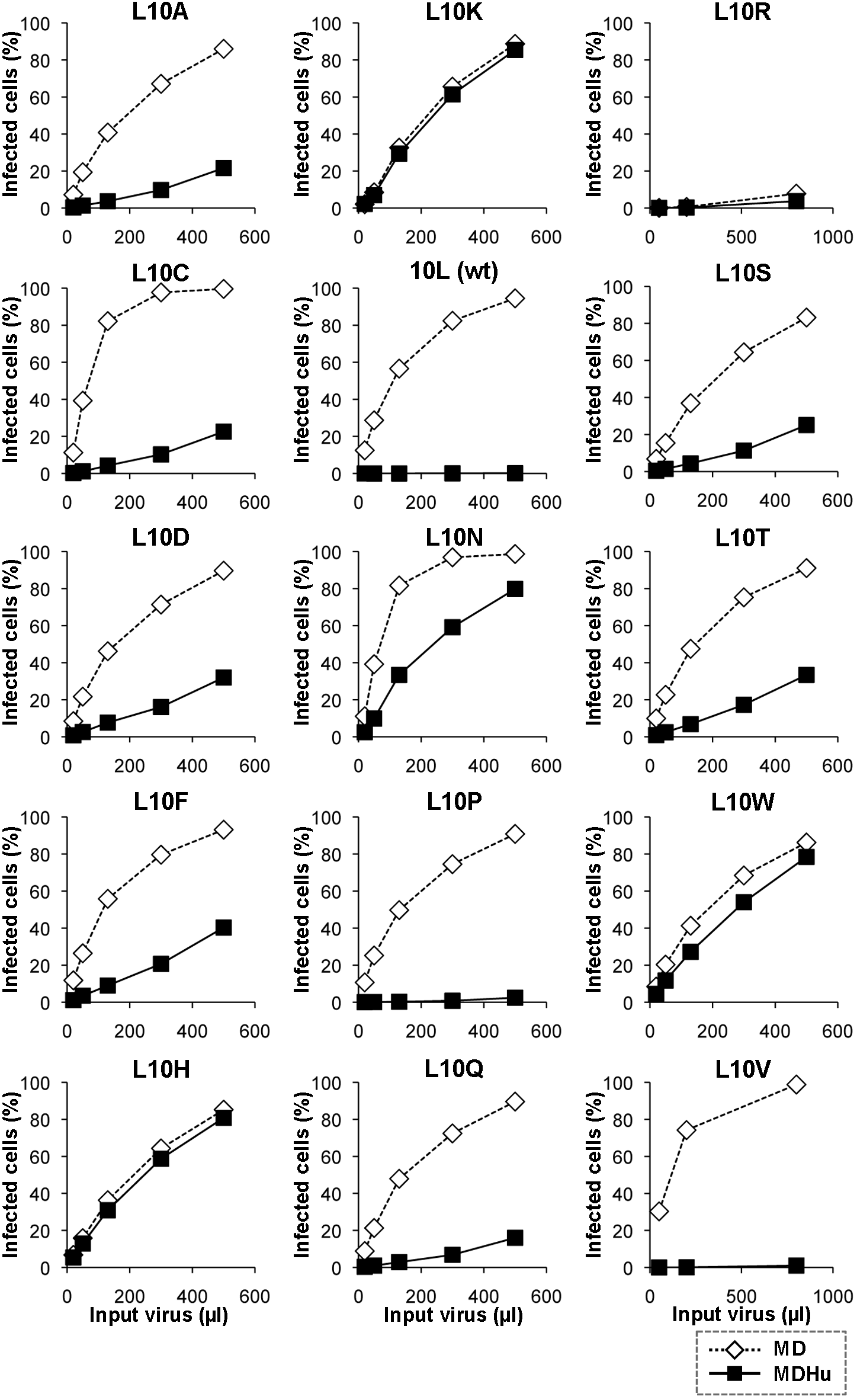

Supplement: Figure S2 — Restriction by rhesus macaque TRIM5α of N-MLV carrying different amino acids at CA position 10. A variety of amino acids were introduced at position 10 of CA by random mutagenesis. Unique isolates were tested for growth in the presence (solid line and filled squares) or absence (dotted line and open diamonds) of rhesus macaque TRIM5α. One representative result from three independent experiments is shown. These data are summarized in Table 2. (TIF) [file ppat.1002011.s002.tif]

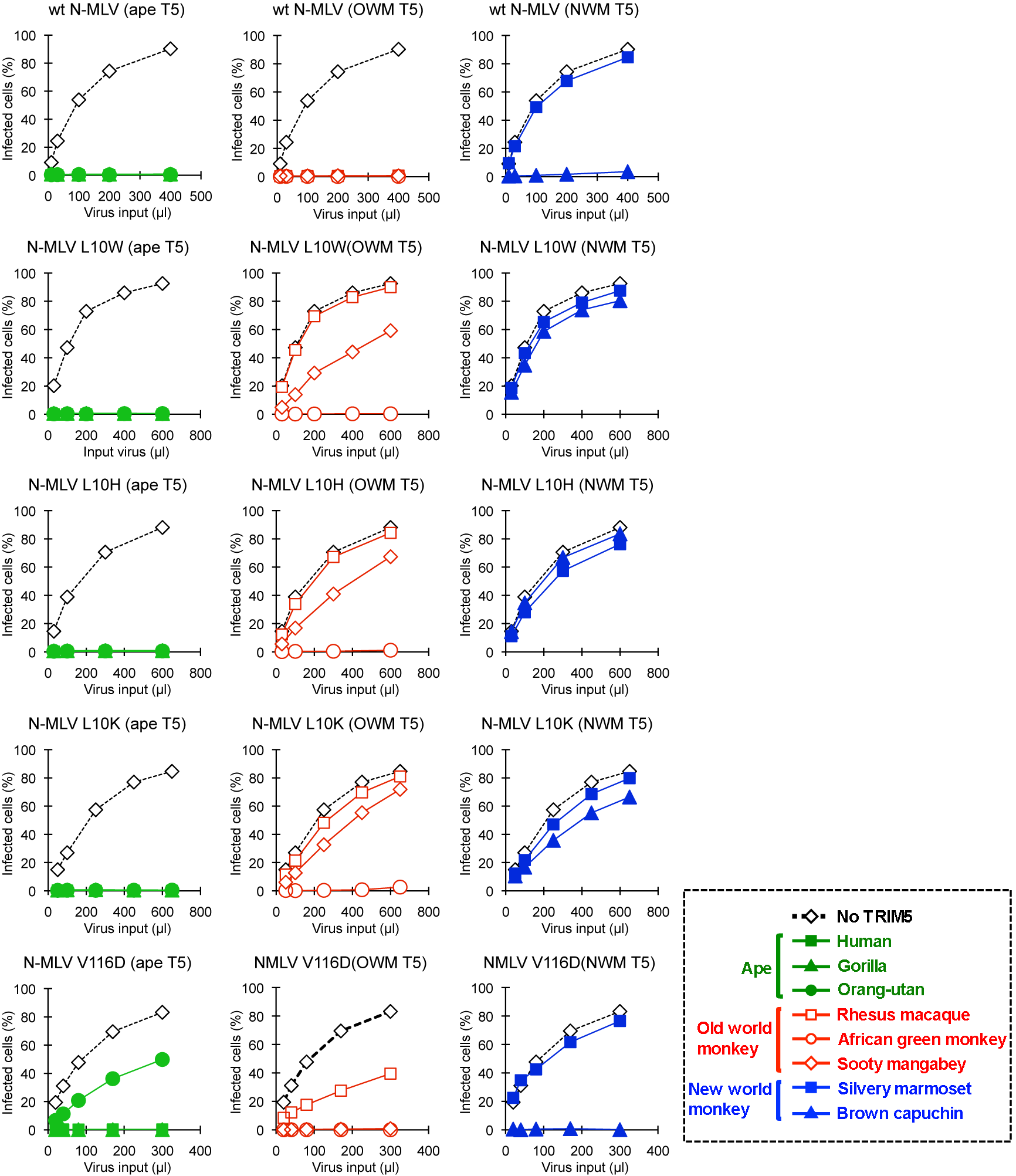

Supplement: Figure S3 — Titration curves of selected N-MLV escape mutants in the presence of various primate TRIM5α. Chimeric TRIM5α constructs expressing the human RBCC domain fused with different primate B30.2 domains [24] were used to test restriction of wild type and L10W, L10H, L10K and V116D escape mutant virus. Titration curves in the presence of ape, old world monkey and new world money TRIM5α are shown in green (left column), red (middle column) and blue (right column), respectively. One representative result is shown from three independent experiments. These data are summarized in Table 3. (TIF) [file ppat.1002011.s003.tif]

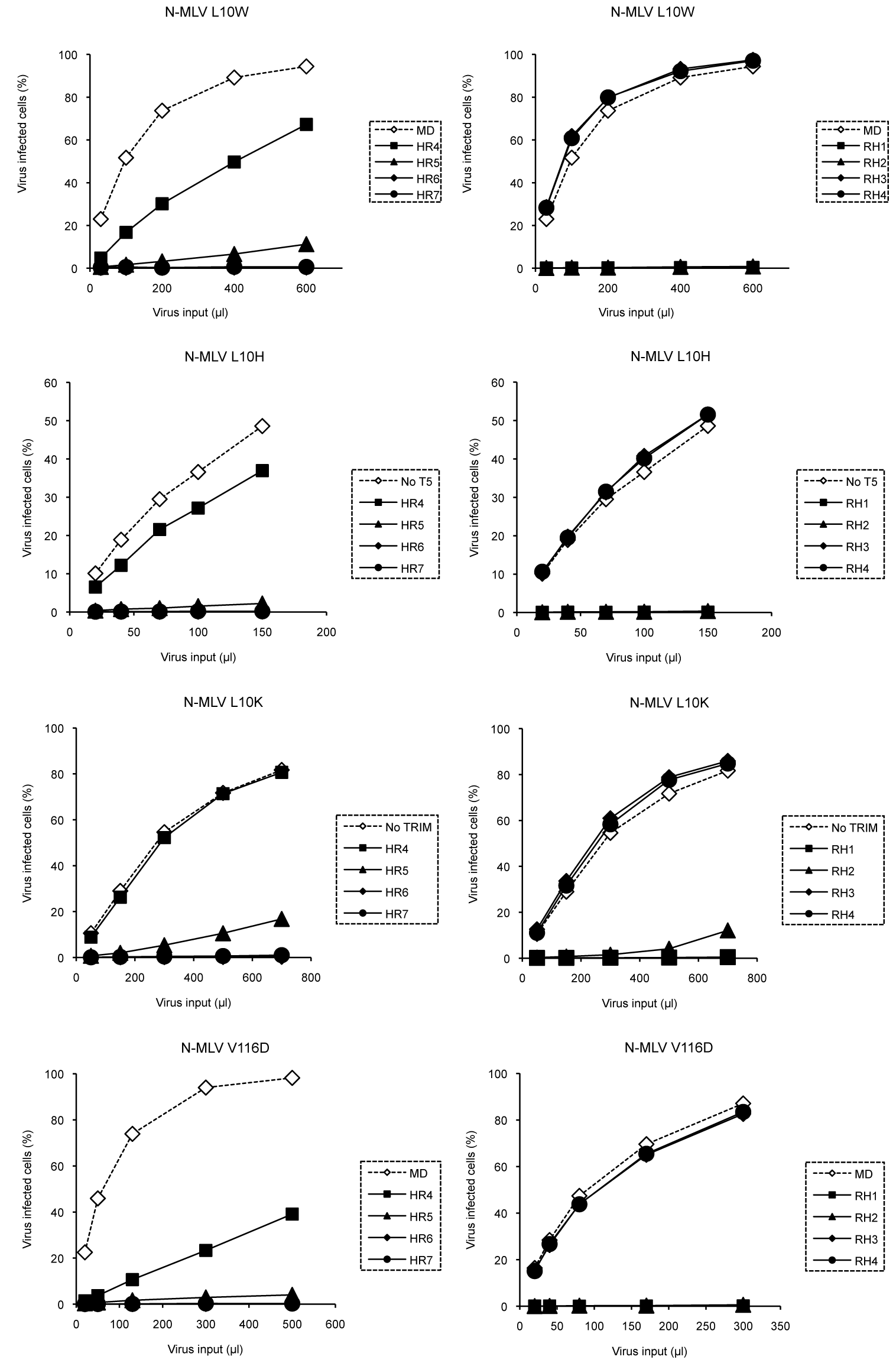

Supplement: Figure S4 — Titration curves of N-MLV escape mutants in the presence of chimeric TRIM5α. Titration curves in the presence of human-rhesus TRIM5α chimeras are shown in the left column and those in the presence of rhesus-human TRIM5α chimera are shown in the right column. One representative result from three independent experiments is shown. These data are summarized in Figure 5. (TIF) [file ppat.1002011.s004.tif]

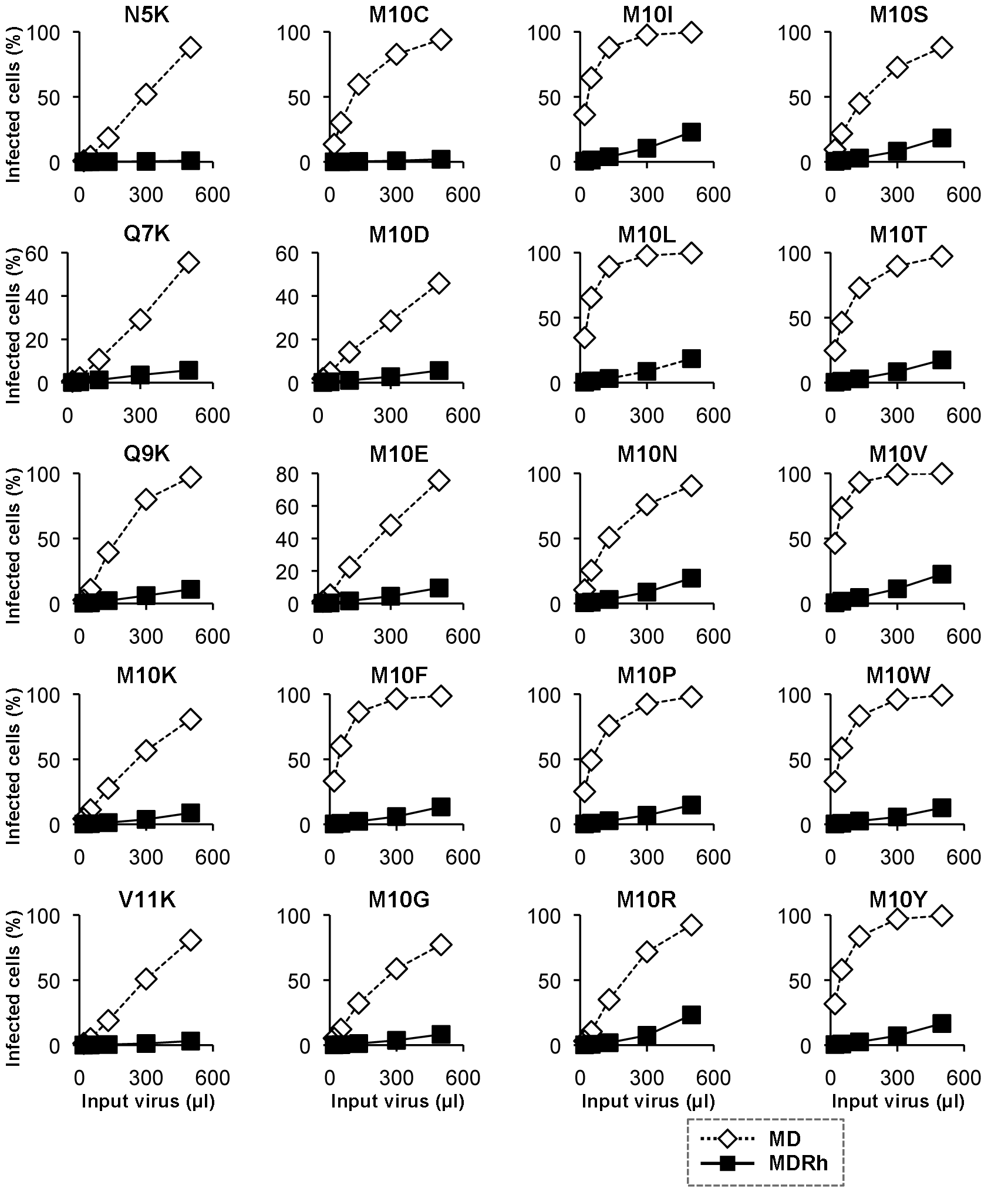

Supplement: Figure S5 — Titration curves of HIV-1 with lysine-substitutions within the CA β-hairpin loop. Lysine substitutions were introduced at amino acid positions 5 through 12 of HIV-1 CA as well as random amino acid mutations at position 10. These were tested for growth in the presence (solid line and filled squares) or absence (dotted line and open diamonds) of rhTRIM5α. Data for the I6K, G8K and H12K mutants showed much reduced infectivity and are not shown. One representative result from two independent experiments is shown. (TIF) [file ppat.1002011.s005.tif]

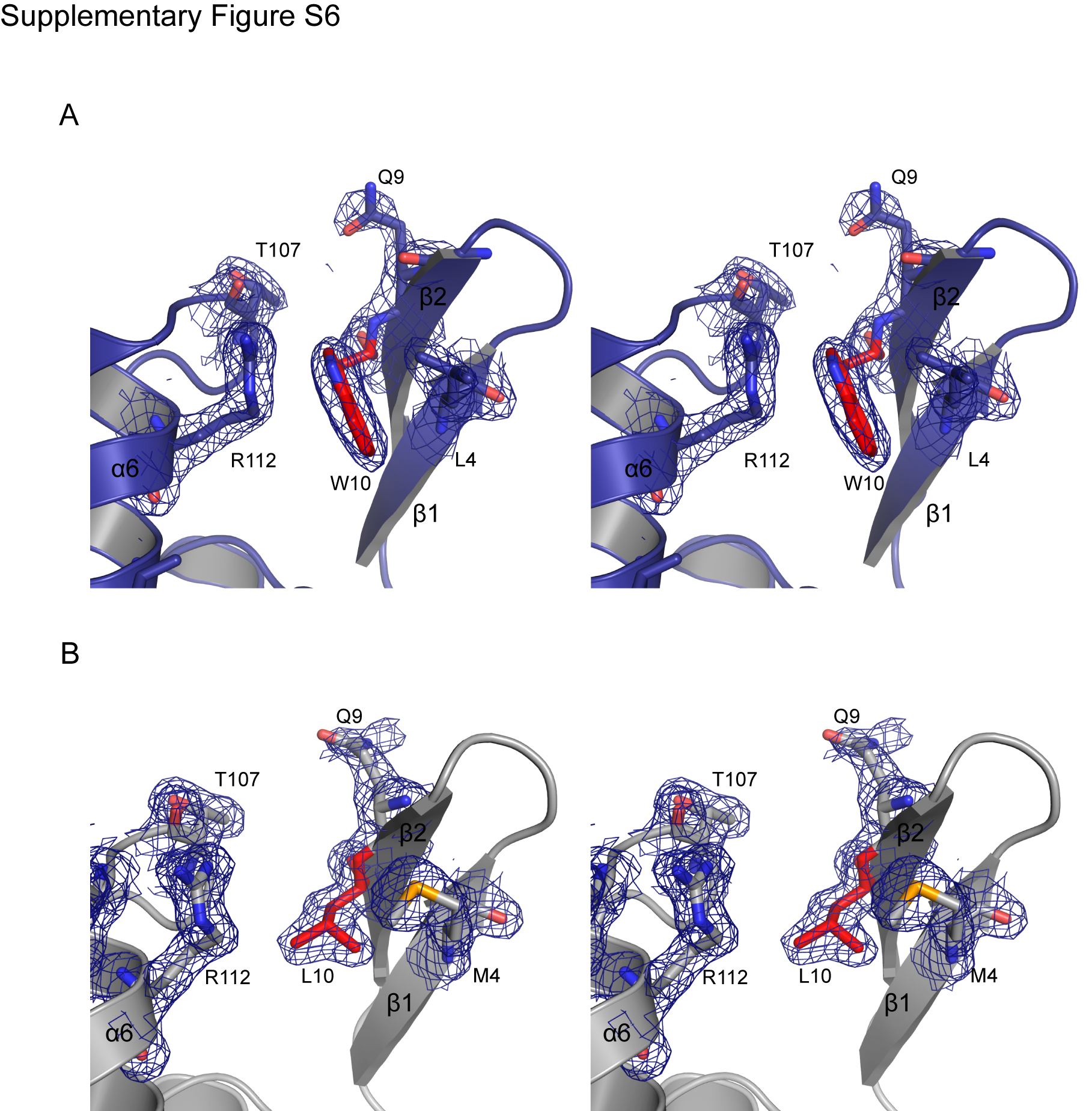

Supplement: Figure S6 — Stereo 2Fo-Fc electron density. Maps contoured at 1.0σ around the region of N-MLV containing either L10 (1U7K) in panel A or W10 (2Y4Z) in panel B are shown. The protein main-chain is shown in cartoon representation, residue side chains are shown in stick representation, L10 and W10 are highlighted in red. (TIF) [file ppat.1002011.s006.tif]

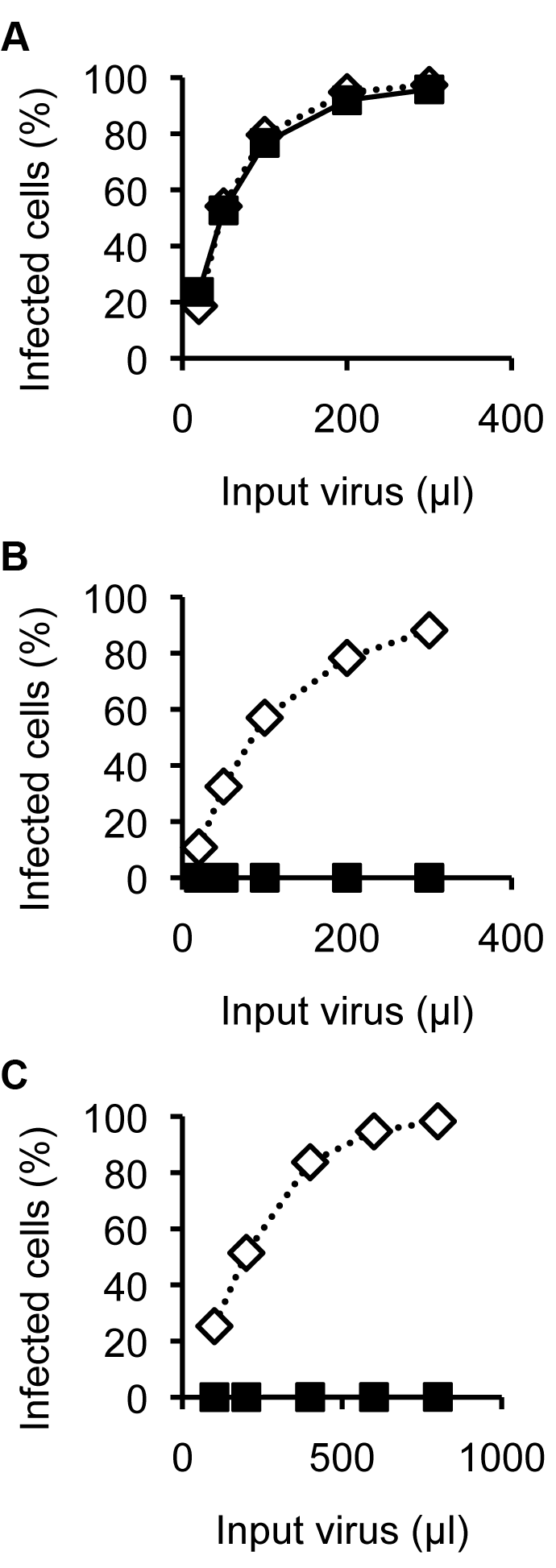

Supplement: Figure S7 — Mutation R112A abolishes relief of TRIM 5α restriction by L10W. Single cycle experiments to examine the effect of the R112A mutation on restriction of wt and L10W N-MLV by TRIM5α. Infection was tested in the presence (filled squares) or absence (open diamonds) of rhTRIM5α. Panel A, N-MLV carrying the L10W mutation; Panel B. N-MLV with R112A; Panel C, N-MLV with L10W and R112A. One representative result from three independent experiments is shown. (TIF) [file ppat.1002011.s007.tif]

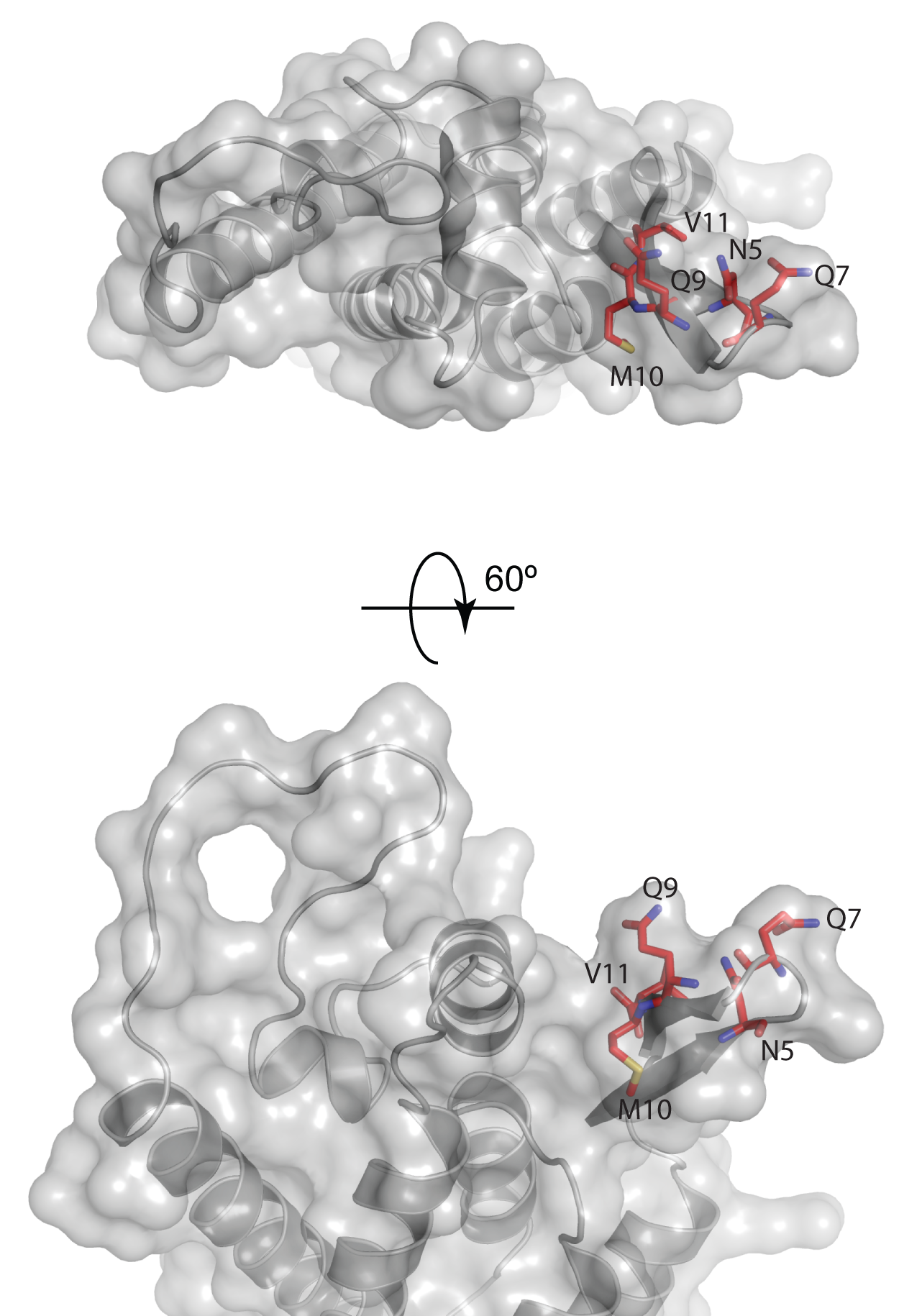

Supplement: Figure S8 — The β1-β2-α6 cleft in HIV-1 CA-NtD. Side (lower) and top (upper) views of the HIV-1 CA-NtD structure are displayed (PDB ID 1M9C). The protein backbone is shown in cartoon representation together with the semi-transparent molecular surface. Residues in the amino-terminal β-hairpin that were mutated in order to look for escape from rhesus TRIM5α restriction are shown in stick representation. The cleft between β1-β2 and helix 5′ at the top of a6 is much wider in HIV than in MLV; cf. Figure 6. (TIF) [file ppat.1002011.s008.tif]
